# Eltrombopag-Induced Cerebral Venous Thrombosis: A Case Report and Literature Review

**DOI:** 10.1155/crh/8955513

**Published:** 2025-10-21

**Authors:** I-Wei Lin, Ken-Hong Lim, Yen-Chang Huang, Meng-Ta Sung

**Affiliations:** ^1^Division of Hematology and Oncology, Department of Internal Medicine, MacKay Memorial Hospital, Taipei City, Taiwan; ^2^Laboratory of Good Clinical Research Center, Department of Medical Research, MacKay Memorial Hospital, New Taipei City, Taiwan; ^3^Department of Medicine, MacKay Medical College, New Taipei City, Taiwan

**Keywords:** anticoagulants, cerebral venous thrombosis, eltrombopag, immune thrombocytopenia purpura

## Abstract

Immune thrombocytopenia purpura (ITP) is initially treated with steroids, but TPO-RAs such as eltrombopag are used for chronic cases. Though effective, eltrombopag has been linked to thromboembolic events, with cerebral venous thrombosis (CVT) being a rare complication. A 20-year-old woman with ITP developed severe headaches, nausea, and vomiting five days after starting eltrombopag. CT scans revealed a dense clot in the right transverse sinus, indicating CVT. Lab data showed elevated platelet counts and D-dimer levels. MRV confirmed CVT, leading to the discontinuation of eltrombopag and initiation of anticoagulant therapy. Recurring thrombocytopenia necessitated further treatment adjustments, including rituximab and cyclosporine, resulting in improved platelet counts and CVT resolution. This case highlighted that CVT is a serious but rare side effect of eltrombopag in ITP patients. Early detection, prompt anticoagulation, and cautious TPO-RA management are crucial for preventing thromboembolic events.

## 1. Introduction

Immune thrombocytopenic purpura (ITP) is an autoimmune disorder characterized by isolated thrombocytopenia, typically presenting with petechiae and mucosal bleeding [1]. Steroids are the first-line treatment for ITP. Thrombopoietin receptor agonists (TPO-RAs), such as eltrombopag and romiplostim, are used to manage chronic ITP after initial treatment failure [2, 3]. While generally safe, TPO-RAs have been associated with thromboembolic events, particularly arterial thromboembolism [4]. Cerebral venous thrombosis (CVT) is a rare manifestation of venous thromboembolism (VTE) associated with eltrombopag therapy. This case report details the clinical presentation, diagnosis, and management of CVT in a young female patient treated with eltrombopag.

## 2. Case Presentation

A 20-year-old woman, diagnosed with primary ITP, presented with gum bleeding and petechiae. No virus infection was detected. Initial autoimmune surveys through enzyme-linked immunosorbent assay (ELISA), including antinuclear factor (ANA), extractable nuclear antigen (ENA) screening, and anti-ds DNA, showed negative findings. Despite dexamethasone therapy for 4 months, her platelet count remained critically low (3000/μL). Eltrombopag (50 mg/day) was initiated after steroid therapy failure.

**Figure 1:**
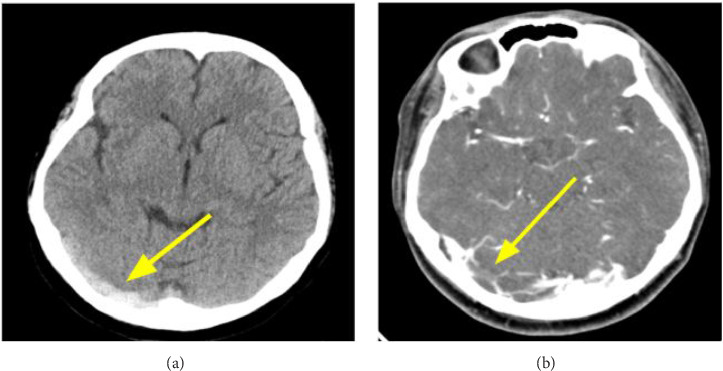
(a) Noncontrast-enhanced computed tomography of the head showing dense clot sign of right transverse sinus (yellow arrow). (b) Computed tomography venography showing small focal filling defects in the right transverse sinus, suspicious of thrombus (yellow arrow).

Five days postinitiation of eltrombopag, the patient developed severe headaches, nausea, and vomiting, prompting an emergency department visit. Initial noncontrast brain CT was unremarkable, but a subsequent CT scan revealed a dense clot sign in the right transverse sinus, suspecting CVT (Figure 1(a)). Elevated platelet counts (589,000/μL) and D-dimer levels (1090 ng/mL) were noted. Coagulopathy and autoimmune disease work-ups were unremarkable.

**Figure 2:**
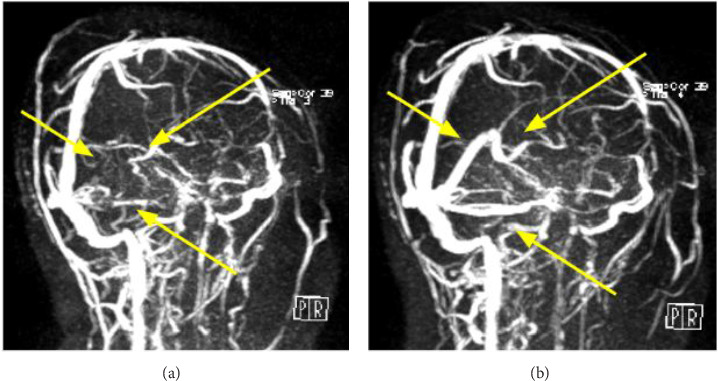
(a) Magnetic resonance venography (MRV) image of initial diagnosis. Arrows (yellow) indicate the filling defects in the vein of Galen, straight sinus, right transverse sinus, and sigmoid sinus, which were compatible with cerebral venous sinus thrombosis. (b) MRV image after anticoagulation therapy revealed that the vein of Galen, the straight sinus, and the transverse sinus presented patency, which indicated reperfusion of the cerebral venous sinuses.

Follow-up magnetic resonance venography (MRV) confirmed CVT with filling defects in the vein of Galen, straight sinus, and right transverse and sigmoid sinuses 2 days later (Figure 2(a)). Eltrombopag was discontinued, and anticoagulant therapy with enoxaparin (1 mg/kg) twice daily for 5 days, followed by edoxaban 60 mg once daily, was initiated. The patient's symptoms improved without neurological sequelae.

Thrombocytopenia (platelet count: 7000/μL) recurred 16 days after discontinuation of eltrombopag. Owing to the risk of bleeding, edoxaban was stopped after 12 days of use, and resumption of ITP treatment, such as rituximab, was considered. Under Taiwan's National Health Insurance (NHI) system, rituximab is not reimbursed and requires self-payment. After discussion, the patient was informed of the risk of recurrent venous thrombosis but nevertheless chose to restart eltrombopag. We started with a lower dose of eltrombopag (25 mg/day). Due to a poor response (platelet count: 10,000–20,000/μL), the dose was increased to 50 mg/day 2 weeks later. The platelet count normalized (317,000/μL) within 7 days after dose escalation.

Severe headaches recurred and led to another ED visit. Brain CT venography revealed small focal filling defects in the right transverse sinus, indicative of CVT (Figure 1(b)). Eltrombopag was again discontinued, and enoxaparin therapy for 3 days, followed by oral edoxaban, was reinitiated.

However, the platelet count fell again. Edoxaban was only prescribed for 3 days and held due to severe thrombocytopenia (6000/μL). The patient finally agreed to use rituximab at her own expense. Rituximab (500 mg) was administered twice during this hospitalization for ITP treatment. MRV after 2 weeks of anticoagulation therapy, 2023 (Figure 2(b)), showed complete resolution of CVT. Cyclosporine (50 mg) and mycophenolate mofetil (500 mg twice daily) were added to elevate the platelet count. By January 2024, the platelet count improved to 168,000/μL, and the patient remained in remission without neurologic sequelae.

## 3. Discussion

TPO-RAs such as eltrombopag and romiplostim have revolutionized the management of ITP, showing efficacy in raising platelet counts in 50%–90% of patients [5]. However, these medications are associated with an increased risk of thromboembolic events. The EXTEND study revealed that approximately 6% of ITP patients treated with eltrombopag experienced thromboembolic events, primarily deep vein thrombosis (DVT) and ischemic stroke [4]. Although it is rare, CVT has been identified as a significant complication of eltrombopag therapy, as illustrated by our case report and systematic review.

We identified nine full-text English case reports or case series related to eltrombopag and CVT in PubMed, which are summarized in Table 1. Most patients were young to middle-aged females, frequently presenting with headaches, a common symptom of CVT. The majority of these cases involved elevated platelet counts and higher doses of eltrombopag (over 50 mg/day). The diagnosis was often confirmed via MRV, which showed filling defects in cerebral sinuses. Management typically involved discontinuing eltrombopag and initiating anticoagulant therapy with enoxaparin or low-molecular-weight heparin, followed by oral anticoagulants. Most patients achieved neurological recovery, although one fatality due to intraparenchymal hemorrhage during enoxaparin treatment was reported. Clinicians should maintain a high index of suspicion for CVT in ITP patients treated with TPO-RAs who present with neurological symptoms such as headache, nausea, or vomiting. Although most patients have favorable outcomes, CVT can lead to severe complications such as seizures, coma, or even death [6–13].

Early detection is crucial to preventing severe complications. Brain CT scans can quickly provide diagnostic indications, with specific signs such as the dense clot sign and empty delta sign. However, MRV remains the gold standard for diagnosing CVT, directly showing the filling defects in the cerebral venous system [6–11]. Discontinuing eltrombopag and initiating anticoagulant therapy are essential management steps. Enoxaparin or heparin, followed by oral warfarin or nonvitamin K antagonist oral anticoagulants (NOACs), has shown good treatment responses for CVT. Our review demonstrated that 78% of patients experienced neurological recovery, although there was one fatality during enoxaparin treatment [10].

The exact mechanism by which TPO-RAs cause VTE remains unclear. Some studies suggest that TPO-RAs increase platelet activity, leading to thrombosis [14]. While others do not support this hypothesis [15–17]. The elevation of platelet counts resulting from effective TPO-RA treatment likely contributes to VTE formation [9, 18]. A rapid increase in platelet counts may also contribute to thrombosis, even if the counts are within the normal range after treatment [7]. In addition, risk factors such as advanced age, a history of thrombosis, and underlying cardiovascular disease further predispose patients to thromboembolic events [5]. In our case, recurrence of CVT following eltrombopag retreatment underscores the necessity of cautious TPO-RA use and vigilant monitoring. When thrombocytopenia recurs, alternative treatments such as rituximab and cyclosporine can help manage platelet counts and prevent thromboembolic events.

Managing CVT in ITP patients is particularly challenging due to the difficulty of maintaining a safe platelet count for anticoagulation. It is generally inappropriate to withhold anticoagulation in ITP patients with thrombosis due to thrombocytopenia, except when the disease is refractory to all other treatments and a minimally acceptable platelet count (≥ 20,000/μL) cannot be achieved [19]. Careful medication adjustment and close monitoring are crucial to avoid further complications. The decision to rechallenge eltrombopag is controversial. Our patient had CVT recurrence after retreatment with eltrombopag, which revealed a strong correlation between CVT and eltrombopag. In contrast, two case reports continued eltrombopag along with continuous anticoagulation therapy without recurrence of CVT, suggesting that this approach may be feasible [7, 12].

Prevention strategies for CVT in ITP patients with TPO-RA have been suggested in some case reports. In Asian populations, a lower starting dose of eltrombopag (25 mg/day) with close follow-up is recommended. In clinical practices in India, aspirin 75–100 mg is administered once daily to patients who achieve a complete response (platelet > 100,000/μL) with eltrombopag to prevent CVT [10]. However, there is no solid evidence to support any prevention strategies for VTE in ITP patients treated with TPO-RAs yet. Assessment of bleeding risk to prevent medication intervention in low bleeding risk patients is another way to reduce medication side effects. Previous available platelet function assays (PFAs) are affected by platelet counts, which cannot measure platelet function accurately in ITP patients. A new viscoelastometry test, Sonoclot coagulation and platelet function analyzer, remains unaffected by the platelet counts [20]. The information from Sonoclot may help in making therapeutic decisions in patients with acute ITP. In lower bleeding risk patients, observation rather than TPO-RA use could be considered to prevent its complications.

CVT is a rare but serious complication of eltrombopag in ITP patients. Early detection, prompt anticoagulation, and cautious management of TPO-RAs are crucial to prevent potentially life-threatening thromboembolic events. The decision to rechallenge eltrombopag should be carefully considered, and thorough thrombophilia work-ups are necessary for ITP patients with thromboembolic events. Prevention strategies, such as starting with lower doses of eltrombopag and using aspirin in patients achieving a complete response, may reduce the risk of CVT, though more evidence is needed to support these approaches.

## 4. Conclusion

CVT is a serious but rare side effect of eltrombopag in ITP patients. Early detection, prompt anticoagulation, and cautious TPO-RA management are crucial for preventing thromboembolic events.

## Figures and Tables

**Table 1:** Comparison of case reports of CVT in ITP patients treated with eltrombopag.

Sex Age	Underlying disease	Initial ITP treatment	ITP duration	Eltrombopag duration dose and duration	Initial presentation of CVT	Platelet level (10^3/μL) before eltrombopag use	Platelet level (10^3/μL) at CVT diagnosis	Velocity of platelet elevation (10^3/μL/day)	Autoimmune screen	CVT diagnostic tool	Thrombosis areas	Treatment of CVT	Eltrombopag continuation	ITP salvage treatment	Outcome	Reference
Female 20 years/o	No underlying disease	Steroid, chloroquine	1 year and 8 months	10 days	Headache	74	589	51.5/day	APL: negative	MRV	Vein of Galen, straight sinus, right transverse sinus, right sigmoid sinus	Enoxaparin 60 mg BID SC, followed by edoxaban 60 mg QD	Discontinue	Rituximab 500 mg fixed dose × 2, cyclosporine 50 mg and mycophenolate mofetil 500 mg twice daily	Complete neurologic recovery MRV: thrombosis resolved	Our case
Female 49 years/o	N/A	Steroid, rituximab	1 year	50 mg for 10 days	Headache Seizure	N/A	48	N/A	APL: negative	MRV	Left transverse sinus Superior sagittal sinus	Low-molecular-weight heparin, followed by rivaroxaban 15 mg QD for 21 days and then 20 mg QD	Discontinue	N/A	Complete neurologic recovery MRV: thrombosis resolved	[5]
Female 55 years/o	N/A	Prednisone, romiplostim, IVIG	18 years	25 mg for 7 days, then 50 mg for 6 days	Headache Nausea Vomiting	3	124	9.31/day	APL: negative	MRV	Right jugular vein Right transverse sinus Right sigmoid sinus	Heparin, followed by warfarin	Keep eltrombopag 50 mg QD	Prednisone 10 mg QD, eltrombopag 50 mg QD	Complete neurologic recovery	[6]
Female 34 years/o	N/A	Steroid	3 years	25 mg for 2 weeks, then 50 mg for 6 weeks	Headache	6	658	11.64/day	N/A	MRV	Right sigmoid sinus	Enoxaparin, followed by warfarin	Discontinue	Prednisone 10 mg QD	Complete neurologic recovery and discharge MRV: thrombosis resolved	[7]
Female 39 years/o	Diabetes mellitus	N/A	Over 1 year	N/A	Headache Nausea Vomiting	N/A	32	N/A	APL: Weak + of anticardiolipin IgM suspected Sjogren's syndrome	Computed tomography venogram (CTV)	Right transverse sinus Superior sagittal sinus Left sigmoid sinus	Heparin, followed by warfarin	Discontinue	Steroids (intravenous dexamethasone 40 mg × 4 days) and intravenous immunoglobulins (IVIGs × 2 days; change to use hydroxychloroquine)	Symptoms relief - > become alert MRV: thrombosis resolved	[8]
Female 27 years/o	N/A	Dexamethasone 40 mg, romiplostim	6 months	50 mg for 4 weeks, then 75 mg for 16 weeks	Headache Projectile vomiting	36 (after 8 weeks of eltrombopag 50 mg/day)	291	4.55/day	APL: negative	Angiography	Left sigmoid sinus	Enoxaparin 1 mg/kg BID	Discontinue	N/A	Intraparenchymal hemorrhage after enoxaparin expired	[9]
Female 36 years/o	N/A	Intravenous immunoglobulins, steroids, and rituximab	11 years	50 mg then 75 mg for 9 months	Headache left-sided weakness	N/A	N/A	N/A	APL: negative	MRV	Superior sagittal sinus	Enoxaparin 60 mg BID, followed by warfarin	Discontinue	Prednisone 10 mg QD	Improvement in left hemiparesis	[10]
Female 36 years/o	N/A	Steroid, splenectomy followed by a course of intravenous immunoglobulins	5 years	50 mg for 3 days	Hemiparesis Headache	5	95	30/day	APL: negative	MRV	Superior sagittal sinus Transverse sinuses	Enoxaparin 0.6 mg BID	Keep eltrombopag 50 mg QD	Keep eltrombopag 50 mg QD	Improvement in left hemiparesis and discharge	[11]
Female 29 years/o	N/A	Dexamethasone 40 mg/day	N/A	50 mg for 4 weeks	Headache Visual blurring nausea	80	212	4.71/day	APL: negative	MRV	Left transverse sinus Left sigmoid sinus Left internal jugular vein	Enoxaparin, followed by edoxaban for 6 months	Discontinue	N/A	Symptom relief and discharge	[12]
Male 75 years/o	N/A	Dexamethasone 40 mg/day	N/A	50 mg for 4 weeks	Headache altered sensorium	15	81	2.35/day	APL: negative	Computed tomography venogram (CTV)	Superior sagittal sinus Left transverse–sigmoid junction	Heparin IV	Discontinue	N/A	Denied craniectomy best supportive care	[12]

## Data Availability

Data sharing is not applicable to this article as no datasets were generated or analyzed during the current study.
